# Toronto Ethnically Diverse face database: a multi-faceted stimulus set

**DOI:** 10.3389/fpsyg.2025.1541546

**Published:** 2025-05-07

**Authors:** Menahal Latif, Nicole Sugden, Maire L. O'Hagan, Margaret C. Moulson

**Affiliations:** Department of Psychology, Toronto Metropolitan University, Toronto, ON, Canada

**Keywords:** face stimuli, face perception, ethnically diverse, emotion expression, eye gaze

## Abstract

Face stimuli are often used in psychological and neuroimaging research to assess perceptual, cognitive, social, and emotional processes. Many available face databases, however, have limited diversity in ethnicity, emotional expression, gaze direction, and/or pose, which constrains their utility to specific contexts. Having a diverse face database can mitigate these biases and may help researchers investigate novel topics that examine the effects of ethnicity on these processes. The Toronto Ethnically Diverse (TED) face database is designed to provide an open-access set of 271 unique White, Black, East-Asian, South-Asian, South-East Asian, Middle Eastern, Multi-racial, and Indigenous adult models. The TED database includes diversity in race, gender, pose, gaze direction, and three emotion variations (neutral, open-mouth happiness, closed-mouth happiness). Validation data of the stimuli based on judgments of the emotional expressions showed high inter-rater reliability and high accuracy as measured by proportion correct and Cohen's kappa scores. Intensity, and genuineness ratings are also presented for each model. The validation results for TED suggest that this face database consists of models displaying their intended emotions with high fidelity. This database will be useful to researchers seeking to study underrepresented groups and to other broad groups of researchers who are studying face perception.

## Introduction

Perceiving human faces is essential for supporting social interaction and maintaining social bonds. Faces convey information regarding the identity, age, sex, race, and emotional state of an individual. They are often the first visual cues available to a perceiver and signal important social information (Little et al., 2011). Studies involving face stimuli investigate high-level vision, identity recognition, emotion perception, social categorization (i.e., age, race, and gender), and trait evaluation (i.e., attractiveness, and/or trustworthiness). Thus, numerous studies in the field of Psychology require face stimuli to investigate novel questions about human interaction. Existing face databases vary across one or more of the following dimensions: race, gender, pose, gaze direction, and emotional expression. Most of these face databases, however, offer diversity in only a few of the above dimensions, limiting researchers from investigating topics that require stimuli that intersect on many dimensions. To date, there are limited publicly available face databases with a substantial number of faces from multiple underrepresented groups coupled with diversity in pose, gaze, and expression. The current study aims to describe the development and validation of the Toronto Ethnically Diverse (TED) face database, a face stimulus set that contains variation along a number of these dimensions, which we hope will facilitate novel research on diverse populations.

Psychological experiments involving face stimuli use face databases that contain predominantly White and/or Western populations. This limits the generalizability of research findings as a growing literature shows that stimuli portraying individuals from diverse ethnic backgrounds can have a profound impact on the perceptions and actions of participants (Conley et al., 2018; Zebrowitz et al., 2010). To examine the perceptual, emotional, and social processes involved when perceiving faces, diverse face databases are needed that reflect the demographics of study participants. As of 2005, there were 28 databases commonly used in face perception research, but only six of them consisted of non-White faces (Gross, 2005). Only two of these face databases consisted of models from various ethnic backgrounds, whereas the other four face databases were strictly limited to East Asian populations. Recently, more databases have been made available containing faces of models from diverse ethnic backgrounds (Coffman et al., 2015; Conley et al., 2018; Dalrymple et al., 2013; Egger et al., 2011; Langner et al., 2010; Pickron et al., 2024; van der Schalk et al., 2011). For example, the racially diverse affective expression (RADIATE) face database is a database designed to reflect the racial demographics in the United States derived from the annual census (Conley et al., 2018). This database contains facial expressions of Black, White, Hispanic, and Asian adults. The Chicago Face Database is a free resource consisting of high-resolution, standardized photographs of Black and White male and female adult models (Ma et al., 2015). Extensions of the Chicago Face Database include CFD-MR and CFD-INDIA which consist of multiracial and Indian male and female adult models (Ma et al., 2021; Lakshmi et al., 2021). The American Multiracial Faces Database (AMFD) is a collection of photographs of self-reported multiracial individuals with accompanying ratings by naïve observers (Chen et al., 2021). The NimStim set of facial expressions (Tottenham et al., 2009) and the 10k US Adult Faces Database (Bainbridge et al., 2013) are databases that contain majority White faces with a smaller number of Black, East Asian, and Latinx faces. The Multi-Racial Mega-Resolution database (MR2) contains 74 images of men and women of European, African, and East Asian descent (Strohminger et al., 2016).

Although there has been a positive change in the ethnic diversity in available face databases, there are still several limitations in existing face databases, which limit the research questions and populations that can be examined. Across face databases, White faces still tend to be the most well-represented; this is especially true for face databases containing variability in other characteristics (e.g., age, pose, emotional expression, gaze direction; Chen et al., 2021; Ebner et al., 2010; Gross, 2005; Langner et al., 2010). Relatedly, even databases that contain ethnic diversity are limited in the ethnicities available. Most diverse datasets focus on East Asian and Black populations. There are very few face databases that contain images of individuals from understudied groups such as South Asian, Southeast Asian, Middle Eastern, Latinx, and multiracial (Chen et al., 2021; Conley et al., 2018; DeBruine and Jones, 2017; Gross, 2005; Pickron et al., 2024). Due to the overrepresentation of White faces and the dominance of male faces in various datasets, only a small number of databases consist of racially diverse female faces. Such biases present in face databases can further perpetuate the social invisibility experienced by women from underrepresented groups (Chen et al., 2021; Gross, 2005; Neel and Lassetter, 2019; Sesko and Biernat, 2010). This is important as targets' race and gender interact to influence social perception and thus have implications for the generalizability of research dominated by White, male faces (Chen et al., 2021).

Several face databases include faces portraying different facial expressions; however, many of these pose limitations due to the number of models and/or the homogeneity of race (Conley et al., 2018). The Facial Recognition Technology (FERET) database presents variations in face pose but only consists of two facial expressions (i.e., neutral and smiling) for the majority White models (Barson, 2003; Gross, 2005). Several other face databases present variation in pose, expression, and other setting characteristics, but contain limited racial diversity. For example, the CAS-PEAL is a database that only contains faces of Chinese models, the CMU PIE database contains faces of majority White models, and the Chicago database only contains photographs of Black and White models (Gao et al., 2008; Ma et al., 2015; Sim et al., 2002). The NIMH-ChEFS face database consists of child models portraying gaze variations (i.e., directed or averted gaze), however, the models are majority White faces (Egger et al., 2011; Gross, 2005). RADIATE is one of the first databases to include individuals from multiple underrepresented groups (i.e., Latinx) portraying 8 emotional expressions; however, this face database does not include variation in pose and gaze direction (Conley et al., 2018). Although many of the above face databases present variations in model attributes, they are limited in the ethnicities represented in the database and/or variations across dimensions like emotion, pose, and eye gaze direction (see Table 1 for a list of the commonly used ethnically diverse face databases and their characteristics).

**Table 1:** Commonly used face databases with images of non-White models available as of march 2025.

**Database**	**Ethnicities**	**Expression**	**No. of expressions**	**Pose**	**Gaze**	**Gender**	**Year**
The Montreal Set of Facial Displays of Emotion	French Canadian, Chinese, and sub-Saharan African	Yes	6	No	No	Yes	2000
CMU Pose, Illumination, and Expression (PIE) Database	White and East Asian	Yes	3	Yes	No	N/A	2000
The MUCT landmarked face database	Cross-section of races (not specified)	Yes	2	No	No	Yes	2008
NimStim	East Asian, Black, Latinx, and White	Yes	8 (open-mouth variations for all except surprise)	No	No	Yes	2009
Radboud Faces Database (RaFD)	White and Moroccan Dutch	Yes	8	Yes	Yes	Yes	2010
Tarr Lab Face Database	East Asian, Black, Latinx, White, and multiracial	Yes	8 (emotions of mostly White and East-Asian faces)	Yes	No	Yes	2012
The Amsterdam Dynamic Facial Expression Set (ADFES)	White and Middle Eastern	Yes	10	Yes	No	Yes	2012
10k US Adult Faces Database	East Asian, Black, Latinx, and White	Yes	Not specified	No	No	Yes	2013
The Chicago Face Database	Black and White	No	1 (neutral)	No	No	Yes	2015
MultiRacial Mega-Resolution database (MR2)	East Asian, Black, and White	No	1 (neutral)	No	No	Yes	2016
Racially Diverse Affective Expression (RADIATE)	East Asian, Black, Latinx, and White	Yes	8 (open-mouth variations)	No	No	Yes	2018
The Chicago Face Database Multiracial (CFD-MR)	Multiracial	No	1 (neutral)	No	No	Yes	2020
The Chicago Face Database India (CFD-INDIA)	South Asian (Indian)	No	1 (neutral)	No	No	Yes	2020
American Multiracial Faces Database (AMFD)	Majority Asian-White and Latinx-White Multiracial faces	Yes	2	No	No	Yes	2020
Diverse Face Images (DFI)	White, South Asian, Latin, Black, East-Asian	No	1 (neutral)	No	Yes	No	2023
Toronto Ethnically Diverse (TED) face database	East Asian, Black, South East Asian, South Asian, Latinx, White, Middle Eastern, Indo-Caribbean, Indigenous, and multiracial	Yes	2 (open-mouth variation for happiness)	Yes	Yes	Yes	2025

This lack of databases containing ethnically diverse faces with variations among emotional expression, pose, and gaze direction leads some researchers to employ computer-generated faces to investigate facial recognition. Computer-generated faces are created by morphing real faces into a composite face representing the race of interest or by generating artificial faces using software algorithms (Chen et al., 2021; Naples et al., 2015; Vetter and Walker, 2011). Chen et al. (2021) conducted a systematic review investigating the literature on multiracial person perception and found that 84% of published studies have relied on computer-generated faces to investigate the recognition of multiracial faces. Using computer-generated faces can allow for increased experimental control and standardization while allowing researchers to ask novel questions about the recognition of faces from underrepresented groups. However, artificially generated faces are highly controlled and can lack the natural variability present within human faces, thereby limiting the ecological validity of research findings (Chen et al., 2021; Gross, 2005). Additionally, real face images as compared to artificial images can elicit different racial categorizations, as well as divergent ratings on dimensions of trustworthiness, competence, and aggression (Balas and Pacella, 2017; Naples et al., 2015; Vetter and Walker, 2011). Artificial faces are also more poorly remembered than real faces. This has been attributed to the frequent exposure to real faces, which contributes to an out-group disadvantage for the memory of artificial faces (Balas and Pacella, 2017). In sum, these findings suggest that the artificiality of computer-generated faces can minimize the variability associated with identity characteristics leading to altered perceptions of faces, thereby making it less ideal for researchers to employ computer-generated faces to study ethnically diverse populations (Chen et al., 2021).

The limitations of existing face databases and computer-generated faces suggest there is still a need for face databases that provide ethnic diversity as well as variation along other dimensions, like emotional expression, pose, and gaze direction. The goal of the current study was to create and validate a large database consisting of photographs varying in gender, race, pose, expression, and gaze direction. The Toronto Ethnically Diverse (TED) face database is a collection of 271 faces of real individuals from diverse racial backgrounds that have been rated on valence, intensity, genuineness, and accuracy of emotional expressions. It is composed of faces of adult models from multiple ethnic backgrounds (i.e., East Asian, Black, Southeast Asian, South Asian, Latinx, White, Middle Eastern, Indo-Caribbean, Indigenous, and multiracial), the majority of whom are women. Faces vary in pose (frontal, three-quarters, profile, chin up, chin down), eye gaze (open, closed, gaze left, gaze right, gaze up, gaze down), and emotional expression (neutral, open-mouth happiness, closed-mouth happiness). Thus, the TED face database improves upon some limitations in pre-existing datasets and can facilitate novel studies that use faces to investigate perceptual, emotional, and social processes by providing a large, standardized face database containing variations of emotion, gaze, and pose in ethnically diverse models.

## Method

### Database development

Models were recruited from a large public university in Canada and the surrounding community. They participated in the study in exchange for either partial course credit or compensation of $15 for their participation. The study ad was posted online and specified that individuals must be 18 years of age or over to participate. Please note that we informed volunteers that their photographs would be shared with others for research purposes without any accompanying personal information. Participants who opted out of sharing their photographs were welcome to complete the study without their data being recorded.

Our 271 volunteers (*M*age = 21.39, *SD* = 5.3; 199 women, 72 men) consisted of ~5% Black, 9% East Asian, 2% LatinX, 3% Middle Eastern, 20% South Asian, 5% South East Asian, 5% Indo Caribbean, 46% White, 7% Multi-racial, and < 1% Indigenous. Ethnicity was self-reported based on the census categories of the 2014 Canadian census. Table 2 shows the demographic characteristics of the models in this face database.

**Table 2:** Stimuli demographic information.

**Demographic characteristics**	
**Age**	**Years**
Range	17–61
Mean	21.39
**Gender**	**Frequency**
Men	72
Women	199
**Ethnicity**	**Frequency**
Black	13
	12 women
	1 man
East Asian	24
	17 women
	7 men
LatinX	5
	4 women
	1 man
Middle Eastern	7
	6 women
	1 man
South Asian	53
	33 women
	20 men
South East Asian	13
	10 women
	3 men
Indo Caribbean	13
	10 women
	3 men
White	124
	90 women
	34 men
Multiracial	18
	16 women
	2 men
Indigenous	1
	1 woman

Ethnicity information for five models was missing.

#### Procedure and measures

Volunteers read and signed a photo release form permitting the use of the photographs for academic research and educational purposes. Volunteers also completed a demographic questionnaire, including questions about age, ethnic background, country of birth, previous countries of residence, and gender.

Prior to being photographed, models were asked to remove any accessories that would visually separate them from the other models (e.g., glasses, headbands, hats). Models were photographed against a white wall and draped with a black scarf to hide clothing and reduce any potential reflected hues on the models' faces.

**Figure 1:**
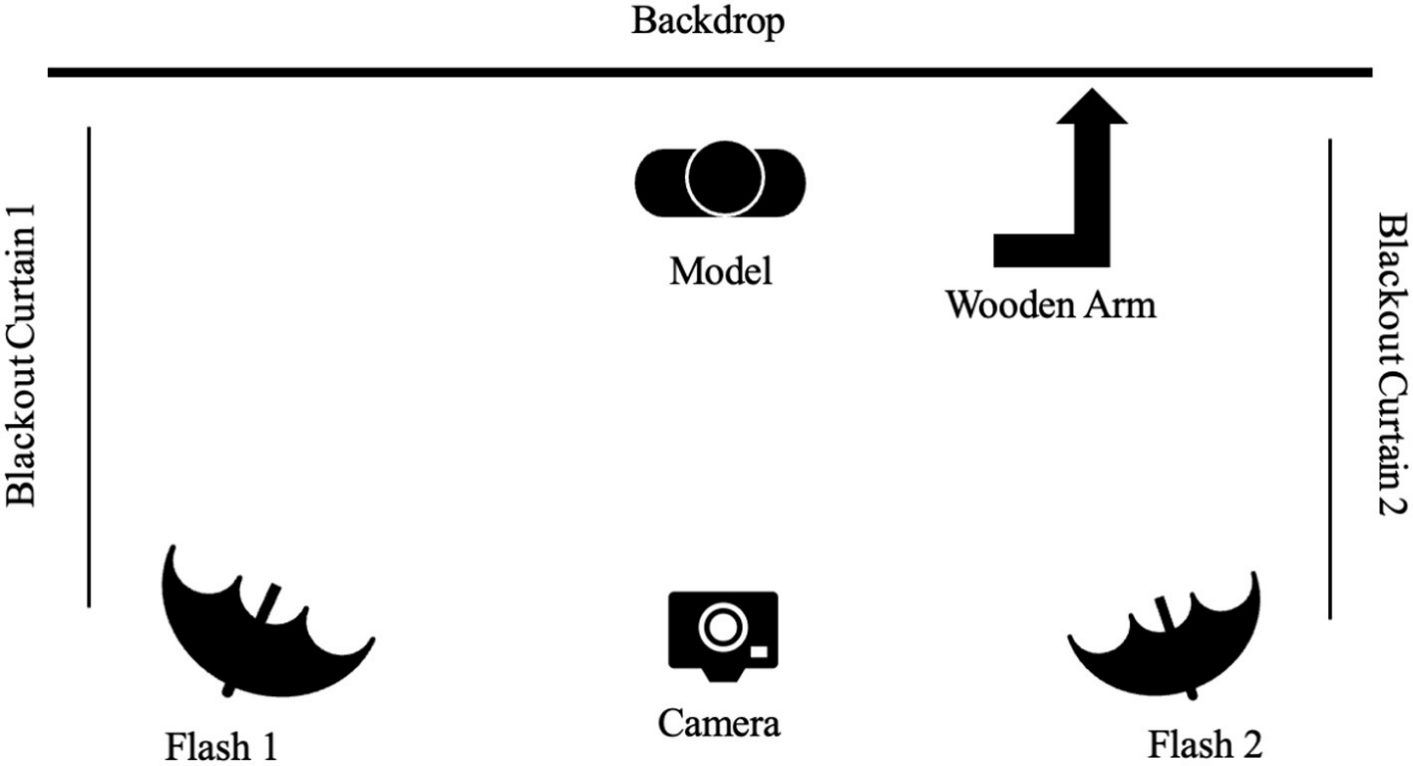
Technical setup of the photoshoot.

Full color photographs were taken with a EOS Rebel T3i camera (affixed to a tripod) and photoshoot lights (placed behind the camera). Participants sat on a stool in front of a white background while facing the camera. A wooden arm with a marker, to align the model's face, was fixed to the closest wall. Research assistants were instructed to swing the arm out to 90 degrees and align the center marker with the eyes of the model before taking the photograph. This alignment was specific for each pose. For example, for the chin-up pose research assistants were asked to align model's bottom lip with the marker on the wooden arm before taking the photograph. See Figure 1 for a diagram of the setup of the photoshoot.

Volunteers were photographed expressing a neutral expression and two happy expressions: an open- and closed-lip smile. Frontal, chin up, and chin down photographs were taken of the models. Photographs were taken of the models head-on, at a three-quarter angle, and of their profile. Further, models posed various gaze conditions: eyes open looking forward, eyes toward the left, eyes toward the right, eyes closed, eyes up, and eyes down. However, not all gaze conditions were fulfilled for each emotion, nor head position. Thus, across dimensions of emotion, head pose, and eye gaze, the number of photographs for each model ranged between 38 and 46 photographs.

To obtain representations of happy and neutral emotional expressions, we asked volunteers to pose the expressions in a way that felt natural to them. These “free posed” expressions offer an alternative to both posed facial expressions (in which participants are asked to move facial muscles in particular ways) and spontaneous facial expressions (expressions that occur in response to natural events). To achieve free-posed expressions, volunteers were asked to think of a time in their life that they felt either neutral or happy and practiced displaying the emotion in a mirror. To further help volunteers display the intended emotion, the experimenter read scenarios that were intended to elicit the specific emotion. Volunteers were not given feedback on their emotional expressions and the experimenter refrained from explaining how to pose (see Supplementary material on Open Science Framework [OSF]). Participants were given as much time as needed until they were ready to have their photo taken. To achieve the eye gaze and pose variations, participants were given the following instructions: now please [turn your head to the right/look to the top-right corner], still expressing [closed-mouth happiness] as naturally as possible. Once the participant was ready, one photograph was taken per condition.

**Figure 2:**
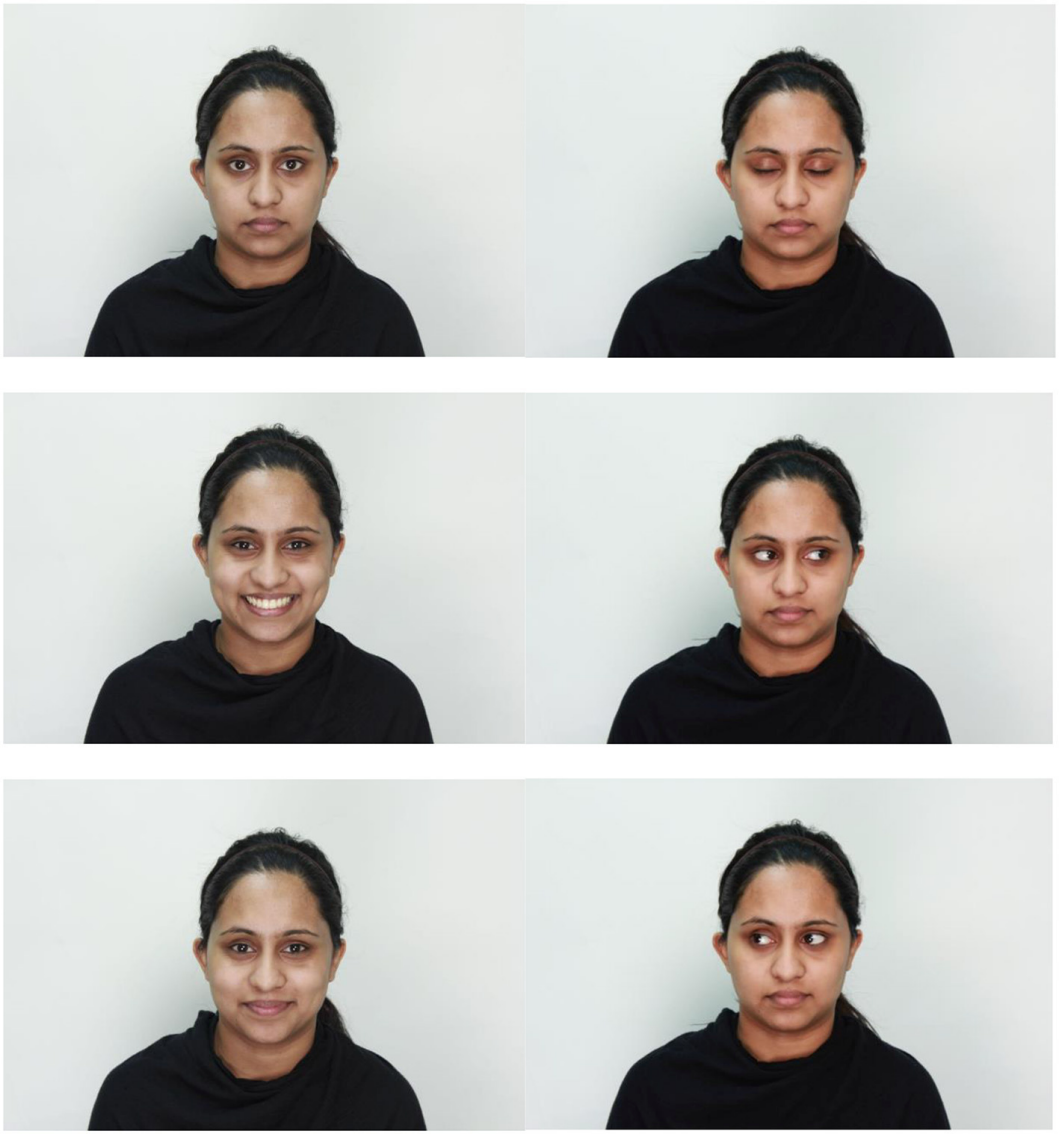
Example stimuli from Model 015. Top left image shows the model with a neutral expression in frontal pose and with open eyes. Top right image shows the model with a neutral expression in frontal pose and with closed eyes. Middle left image shows the model expressing open-mouth happiness in a frontal pose and with open eyes. Middle right image shows the model with a neutral expression in frontal pose and with gaze direction left. Bottom left image shows the model expressing closed-mouth happiness in a frontal pose and with open eyes. Bottom right image shows the model with a neutral expression in frontal pose and with gaze direction right.

Using Adobe Photoshop (v2014.0.1), all photographs were resized to 5,184 × 3,456 pixels, such that the target face and core facial features were approximately centered in the image. Photographs were then cropped in a headshot format using the rectangular cropping tool. Example stimuli are displayed in Figure 2.

### Stimulus validation

Only front facing photographs were used in the current validation study. We obtained stimulus ratings from 502 undergraduate students at Toronto Metropolitan University who received partial course credit for their participation (*M*age = 19.45, *SD* = 3.21). The sample included 89 men (17.8%), 407 women (81.1%), two gender-fluid individuals (0.4%), and four non-binary individuals (0.8%). According to their self-reported ethnicity, participants were East Asian (*n* = 69), Black (*n* = 23), Southeast Asian (*n* = 64), South Asian (*n* = 77), Latinx (*n* = 16), White (*n* = 164), Middle Eastern or North African (*n* = 43), Indo-Caribbean (*n* = 11), and Multiracial (*n* = 32). Three participants did not specify their ethnicity. Our sample goal was set to obtain ~40 raters per face. We determined this goal based on previous research indicating that face-based ratings become stable at about 40 independent observations, or earlier depending on the particular attribute (Coffman et al., 2015). Thus, we ensured that each photograph was rated by at least 40 participants.

#### Procedure

Participants were asked to view and rate the photographs to assess whether they depicted the intended emotional expressions. They completed the survey online through Qualtrics. After providing informed consent, participants were asked to provide demographic information, including their age, gender, ethnic group, country of birth, and, if not born in Canada, the year moved to Canada. Participants were then shown one photograph at a time and were asked to give four ratings. The first question was: “What emotion is this face presenting?” with the following answer choices: Anger, Disgust, Fear, Happiness, Neutral, Sad, Surprise, or None of the listed emotions. The second question was: “What is the valence of the emotion? (Is it a positive or negative emotion?)” with the following options: Negative, Neutral, or Positive. Next, they were asked to “Rate the Intensity of the Emotion” on a scale from 1 (neutral) to 5 (extremely intense). Lastly, participants were asked “How genuine is this emotion?”, which they rated on a scale from 1 (not genuine at all) to 5 (very genuine). Each participant rated ~90 photos (i.e., 30 unique identities/models displaying two different emotions with three total variations). This number is an approximation as not all identities had photographs for the full set of emotions. Each set of 30 unique models contained both male and female models of different ethnicities. The photographs were presented in random order. Each face was rated by ~50 raters on average (range: 41–84). Participants' ratings were only included in the analyses presented below if they completed at least 50% of the survey.

Instructions to access the face database and all data files are posted online on the Open Science Framework (https://osf.io/6vdn2/?view_only=14a1fcd1f3dd434bbfe6a4028fd96400). On this site, researchers can find the descriptive statistics for each model (Supplementary Tables on OSF) and a readme file, as well as instructions to contact the Brain and Early Experiences (BEE) Lab at Toronto Metropolitan University at beelab@torontomu.ca to gain access to the images in the database.

## Results

The Toronto Ethnically Diverse (TED) face database includes 271 unique individuals who each posed with several variations of a neutral expression and a happy expression. These variations included pose and gaze direction. For the stimulus validation, only direct gaze frontal view photos were rated. The total number of photographs that were rated is 805 (270 neutral, 268 open-mouth happiness, 267 closed-mouth happiness).

To determine whether emotional expression, ethnicity, or their interaction impacted our other dependent measures, analyzed the descriptive statistics, and then conducted a two-way analysis of variance on valence, intensity, and genuineness ratings. These results are discussed below.

### Inter-rater reliability

First, two measures, proportion correct and Cohen's kappa (Cohen, 1960), were calculated for each of the 805 stimuli, modeled after the analyses of the NimStim Set of Facial Expressions and the American Multi-racial Face Database (Chen et al., 2021; Tottenham et al., 2009). For each stimulus (photograph), proportion correct was calculated by comparing the number of participants who endorsed the correct target expression to the total number of participants who rated that photograph. Though proportion correct is often reported in examinations of facial expression (Ekman and Friesen, 1975; Mandal, 1987; Bieh et al., 1997; Beaupré and Hess, 2005; Wang and Markham, 1999), Tottenham et al. (2009) suggest that Cohen's kappa (Cohen, 1960) may be a better dependent variable for evaluations of face databases since proportion correct does not consider false positives (Erwin et al., 1992). Therefore, kappa scores, a measure of agreement between participants' labels and models' intended expressions adjusted for agreement due to chance, were used to estimate agreement between selected labels and intended expressions. These scores were calculated across models within each survey, independently for open- and closed-mouth conditions. Endorsements of “none of the above” were counted as incorrect.

**Figure 3:**
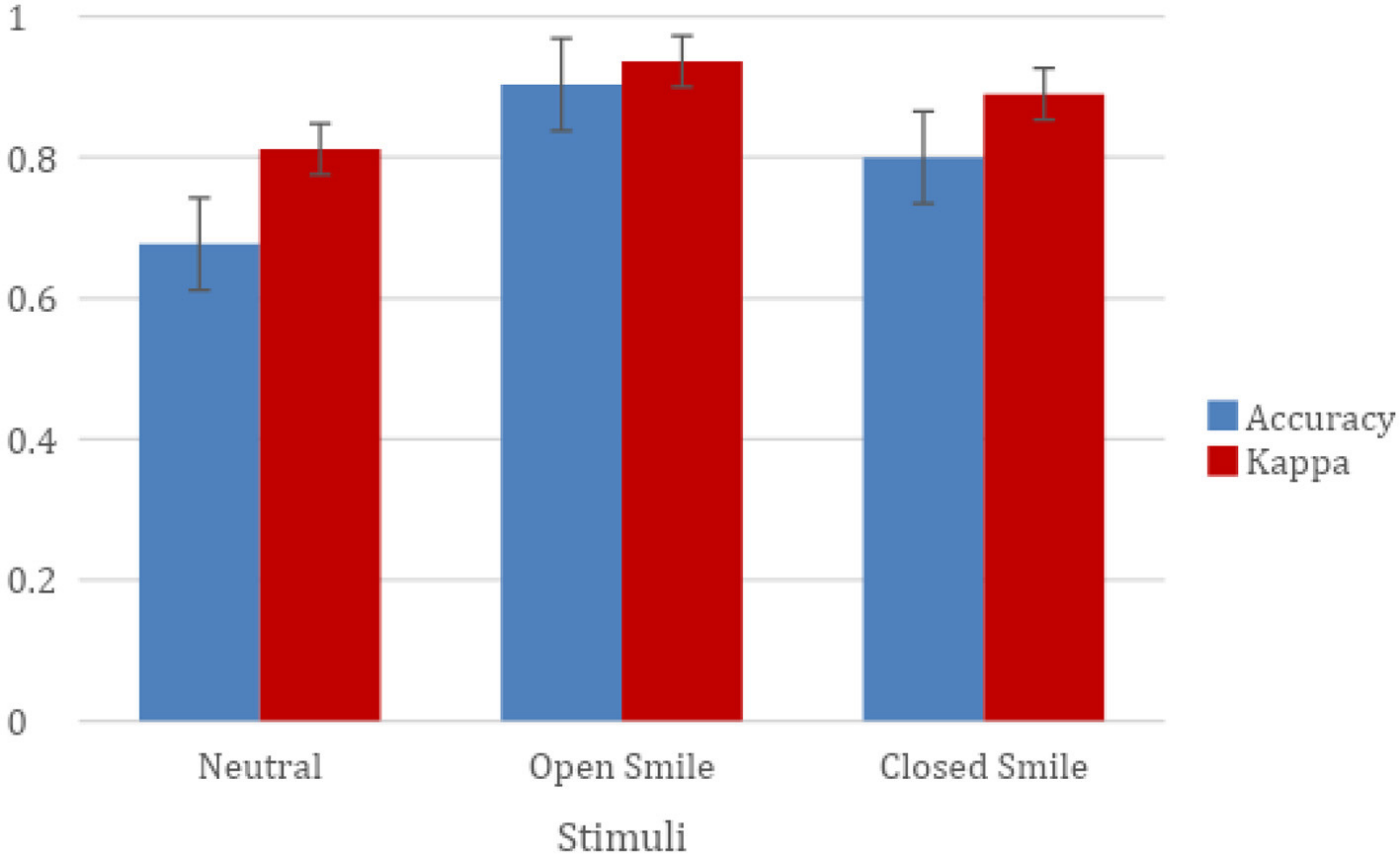
Mean proportion correct and kappa scores for each of the three emotional expressions. Error bars represent standard error of the mean.

**Figure 4:**
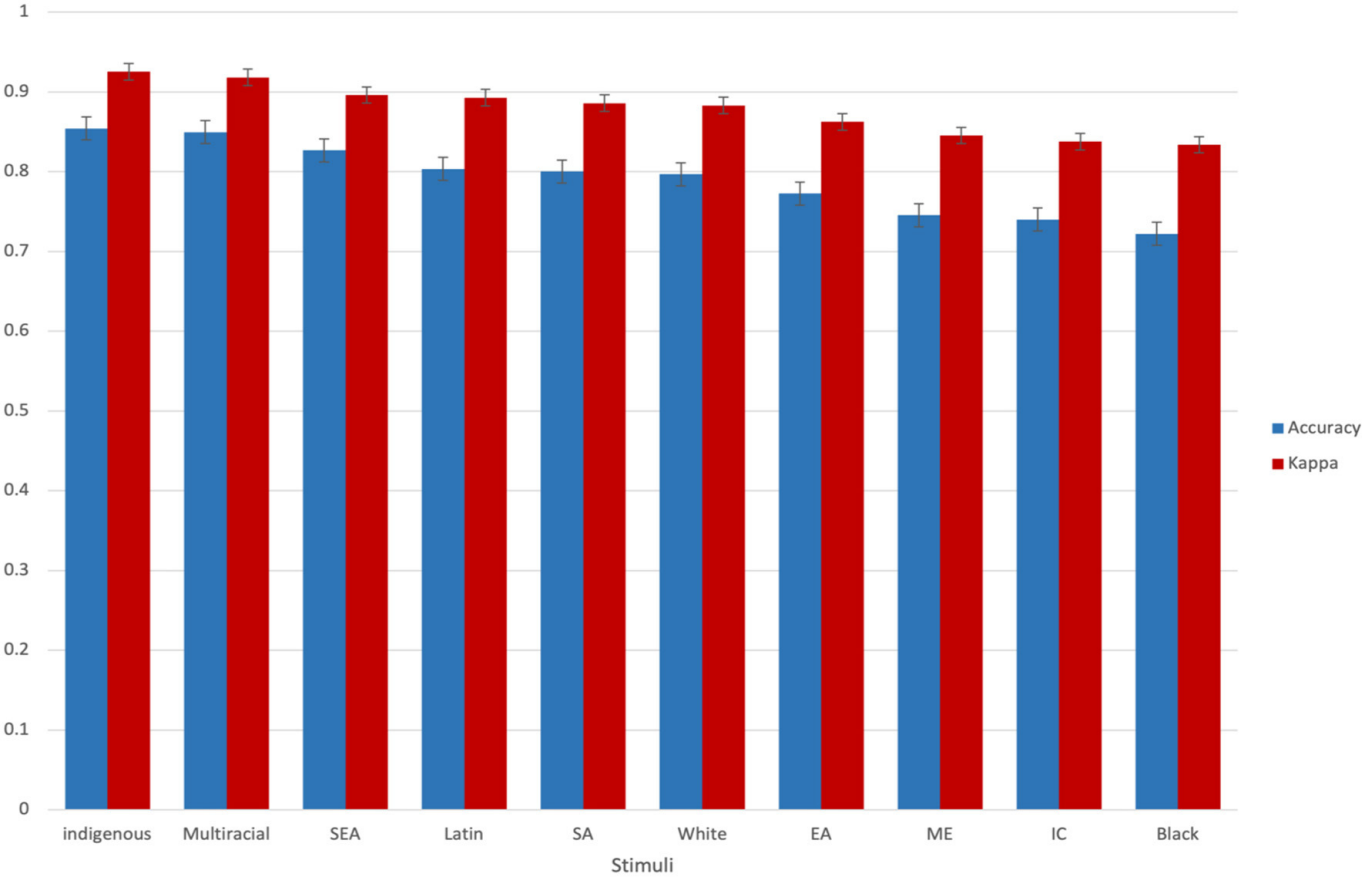
Mean proportion correct and kappa scores for each of the 10 model ethnic groups. Error bars represent standard error of the mean.

Average ratings for each of the three emotional expressions are presented in Table 3 and Figure 3 and average ratings separated by face ethnicity are presented in Figure 4 (see Supplementary Tables on OSF for proportion correct and kappa score for individual photographs and for other descriptive statistics by ethnicity). Overall, proportion correct (Mean = 0.79, *SD* = 0.23, Median = 0.88) and kappa scores (Mean = 0.88, *SD*= 0.14, Median = 0.93) were high, indicating that stimuli accurately conveyed their intended expressions (Landis and Koch, 1977). Kappa scores per model ranged from 0.6 to 1 for 93% of the models (i.e., 251 of the total 271 models), reflecting general agreement between participants' labels and models' expressions, adjusting for agreement due to chance (Cohen, 1960; Landis and Koch, 1977). Of the mean proportion correct scores, 54% (147/271) were above 0.70.

**Table 3:** Descriptive statistics for each of the three emotional expressions.

**Stimuli**	**Measures**	***M***	***SD***	**Range**
Neutral	Accuracy (%)	0.68	0.19	0.07 to 1
	Kappa	0.81	0.12	0.31 to 99
	Valence (−1 to +1)	−0.18	0.25	−0.78 to 0.82
	Intensity (1–5)	1.92	0.23	1 to 2.73
	Genuineness (1–5)	3.14	0.24	2.4 to 4.2
Open smile	Accuracy (%)	0.90	0.19	0 to 1
	Kappa	0.94	0.12	0.31 to 1
	Valence (−1 to +1)	0.86	0.22	−0.38 to 1
	Intensity (1–5)	3.52	0.57	1 to 4.62
	Genuineness (1–5)	3.76	0.57	1 to 4.64
Closed smile	Accuracy (%)	0.80	0.24	0 to 1
	Kappa	0.89	0.13	0.25 to 1
	Valence (−1 to +1)	0.71	0.30	−0.74 to 1
	Intensity (1–5)	2.82	0.51	1 to 3.82
	Genuineness (1–5)	3.44	0.51	1 to 4.46

*N* = 502.

#### Proportion correct

The two-way analysis of variance on the proportion correct scores found a significant effect of emotion, *F*_(2,802)_ = 81.63, *p* < 0.001, *n2* = 0.17. Open-mouth happy faces were rated more accurately (*M* = 0.90, *SD* = 0.19) than closed-mouth happy faces (*M* = 0.80, *SD* = 0.24), *t*_(509)_ = 5.55, *p* < 0.001, and neutral faces (*M* = 0.68, *SD* = 0.19), *t*_(535)_ = 13.94, *p* < 0.001. Closed-mouth happy faces were also rated more accurately than neutral faces, *t*_(502)_ = 6.67, *p* < 0.001. We did not find a significant effect of ethnicity or an interaction between emotion and ethnicity.

#### Kappa scores

Similar to findings for proportion correct, the two-way analysis of variance on the kappa scores found a significant effect of emotion, *F*_(2,802)_ = 68.45, *p* < 0.001, *n2* = 0.15. Open-mouth happy faces (*M* = 0.94, *SD* = 0.12) were rated as having significantly higher agreement than closed-mouth happy faces (*M* = 0.89, *SD* = 0.13), *t*_(532)_ = 4.28, *p* < 0.001, and neutral faces (*M* = 0.81, *SD* = 0.12), *t*_(536)_ = 11.71, *p* < 0.001. Closed-mouth happy faces were also rated as having significantly higher agreement than neutral faces, *t*_(534)_ = 7.23, *p* < 0.001. In contrast to findings for proportion correct, we found a significant effect of ethnicity on kappa scores, *F*_(9,775)_ = 2.31, *p* < 0.050, *n*2 = 0.02. Multi-racial faces (*M* = 0.92, *SD* = 0.08) had significantly higher agreement than South-Asian faces (*M* = 0.89, *SD* = 0.12), *t*_(204)_ = 1.86, *p* < 0.050, White faces (*M* = 0.88, *SD* = 0.13), *t*_(419)_ = 1.81, *p* < 0.050, East-Asian faces (*M* = 0.86, *SD* = 0.17), *t*_(122)_ = 2.19, *p* < 0.050, Middle Eastern faces (*M* = 0.84, *SD* = 0.13), *t*_(71)_ = 3.50, *p* < 0.010, Indo-Caribbean faces (*M* = 0.84, *SD* = 0.18), *t*_(88)_ = 2.83, *p* < 0.010, and Black faces (*M* = 0.83, *SD* = 0.18), *t*_(92)_ = 3.02, *p* < 0.010. Southeast-Asian faces (*M* = 0.89, *SD* = 0.11) had significantly higher agreement than Middle-Eastern faces (*M* = 0.84, *SD* = 0.13), *t*_(56)_ = 1.68, *p* < 0.050, and Black faces (*M* = 0.83, *SD* = 0.18), *t*_(77)_ = 1.80, *p* < 0.050. South-Asian faces (*M* = 0.89, *SD* = 0.12) had significantly higher agreement than Indo-Caribbean faces (*M* = 0.84, *SD* = 0.18), *t*_(188)_ = 1.95, *p* < 0.050, and Black faces (*M* = 0.83, *SD* = 0.18), *t*_(192)_ = 2.25, *p* < 0.050. Similarly, White faces (*M* = 0.88, *SD* = 0.13) had significantly higher agreement than Indo-Caribbean faces (*M* = 0.84, *SD* = 0.18), *t*_(403)_ = 1.97, *p* < 0.050, and Black faces (*M* = 0.83, *SD* = 0.18), *t*_(407)_ = 2.32, *p* < 0.050. We did not find a significant interaction between emotion and ethnicity.

The confusion matrix (Table 4) depicts the average proportion of target and non-target labels endorsed for each expression, revealing patterns of misidentifications. This matrix demonstrates that expressions were rarely identified as “none of the above” (endorsement of “none of the above” across expressions ranged from 0.02 to 0.05). Overall, participants accurately endorsed target labels for each expression. However, faces displaying a neutral expression were most often mislabeled as “sad” or “anger” expressions (endorsement of both “sad” or “anger” was 0.08). Faces displaying an open-mouth happy expression were occasionally identified as neutral or surprise (endorsement of both “neutral” or “surprise” was 0.03) and faces with closed-mouth happy expressions were most often mislabeled as neutral (endorsement of “neutral” was 0.11). These results are consistent with previous face databases (Tottenham et al., 2009; Langner et al., 2010; Conley et al., 2018) that confirm that neutral faces are often mislabeled as sad or anger. Also, the higher identification rate for happy than neutral expressions found in the current study is consistent with previous research (Hare et al., 2005). As well, emotion recognition studies suggest that closed-mouth happy expressions are most often mislabeled as neutral (Beaupré and Hess, 2006; Ekman and Friesen, 1975).

**Table 4:** Confusion matrix depicting proportion of responses for each emotion label (*SD*).

**Photograph**	**Anger**	**Disgust**	**Fear**	**Happiness**	**Neutral**	**Sad**	**Surprise**	**None of the listed emotions**	**N/A**
Neutral	0.08	0.03	0.03	0.05	**0.66**	0.08	0.01	0.05	0.02
	(0.10)	(0.04)	(0.04)	(0.11)	**(0.18)**	(0.10)	(0.03)	(0.04)	(0.02)
Happiness (Open)	0.00	0.01	0.00	**0.89**	0.03	0.00	0.03	0.02	0.02
	(0.01)	(0.02)	(0.01)	**(0.17)**	(0.06)	(0.01)	(0.08)	(0.05)	(0.01)
Happiness (Closed)	0.01	0.01	0.01	**0.79**	0.11	0.02	0.01	0.04	0.02
	(0.02)	(0.03)	(0.01)	**(0.23)**	(0.16)	(0.03)	(0.03)	(0.05)	(0.01)

*N* = 502. The bold values represent average proportion of target labels endorsed for each expression.

### Valence

Participants' valence responses (negative/neutral/positive) were coded as negative = −1, neutral = 0, positive = +1. We found a significant effect of emotion, *F*_(2,802)_ = 167.79, *p* < 0.001, *n2* = 0.76. Open-mouth happy faces were rated as significantly more positive (*M* = 0.86, *SD* = 0.22) than closed-mouth happy faces (*M* = 0.71, *SD* = 0.30), *t*_(496)_ = 6.61, *p* < 0.001 and neutral faces (*M* = −0.18, *SD* = 0.25), *t*_(499)_ = −51.32, *p* < 0.001. As well, closed-mouth happy faces were rated as significantly more positive than neutral faces, *t*_(498)_ = −37.59, *p* < 0.001. We did not find a significant effect of ethnicity on valence ratings or an interaction between emotion and ethnicity.

### Intensity

There was also a significant effect of emotion on intensity ratings, *F*_(2,802)_ = 806.5, *p* < 0.001, *n2* = 0.67. Open-mouth happy faces (*M* = 3.52, *SD* = 0.57) were rated as significantly more intense than closed-mouth happy (*M* = 2.82, *SD* = 0.51), *t*_(527)_ = 14.82, *p* < 0.001, and neutral faces (*M* = 1.92, *SD* = 0.23), *t*_(351)_ = 42.32, *p* < 0.001. Closed-mouth happy faces were also rated significantly more intense than neutral faces, *t*_(370)_ = 26.28, *p* < 0.001. We did not find a significant effect of ethnicity on intensity ratings or an interaction between emotion and ethnicity.

### Genuineness

There was also a significant effect of emotion on genuineness ratings, *F*_(2,802)_ = 121, *p* < 0.001, *n2* = 0.23. Open-mouth happy faces (*M* = 3.76, *SD* = 0.57) were rated as significantly more genuine than closed-mouth happy (*M* = 3.44, *SD* = 0.51), *t*_(526)_ = 6.88, *p* < 0.001, and neutral faces (*M* = 3.14, *SD* = 0.24), *t*_(358)_ = 16.38, *p* < 0.001. Closed mouth happy faces were also rated significantly more genuine than neutral faces, *t*_(379)_ = 8.70, *p* < 0.001. We did not find a significant effect of ethnicity or an interaction between emotion and ethnicity. Table 5 reports the descriptive statistics for each rating dimension collapsed across all stimuli in TED.

**Table 5:** Descriptive statistics for entire face database.

**Measures**	***M***	***SD***	**Range**
Accuracy (%)	0.79	0.23	0 to 1
Kappa	0.88	0.13	0.25 to 1
Valence (−1 to +1)	0.46	0.52	−0.78 to 1
Intensity (1–5)	2.75	0.80	0 to 4.62
Genuineness (1–5)	3.45	0.53	0 to 4.64

*N* = 502.

## Discussion

Face perception is an essential process for day-to-day social interactions. Researchers who are interested in studying face perception and related impression formation processes frequently rely on convenience samples of stimuli. While there are a number of high-quality face databases available to researchers, there are very few with ethnically diverse models that also present variations in emotional expression, gaze direction, and pose. Increasing the number of stimuli can add to the heterogeneity of existing face databases, which will correspondingly improve our field's ability to produce generalizable results and empirically address a broader set of issues (Chen et al., 2021). In this paper, we present the Toronto Ethnically Diverse (TED) face database, a collection of 271 unique models with accompanying ratings by a diverse sample of participants. We provide neutral and smiling versions of faces for researchers to have additional flexibility in their research questions and methods. The TED face database is an open-access resource for researchers interested in psychological processes involving face processing and social perception.

The validation results for TED suggest that this face database consists of models displaying their intended emotions with high fidelity: Mean proportion correct was 0.79 across the entire face database. Similarly, the mean kappa score, which measures agreement between participants' labels and models' intended expressions, taking into account incorrect judgments, was 0.88. This reflects high agreement among participants that stimuli conveyed their intended expressions.

Despite overall high inter-rater agreement, the ethnicity of the face impacted kappa scores for emotion ratings. Indigenous, Multi-racial, South-Asian, Southeast Asian, Latin, and White faces showed the highest kappa scores (i.e., the highest agreement among participants that the stimuli conveyed their intended emotion), with Black and Indo-Caribbean faces showing the lowest kappa scores. Although it is uncertain what the source of these ethnic group differences is, it is important to note that average kappa scores for all ethnic groups were high, ranging from 0.83 to 0.93, in line with existing databases (Conley et al., 2018; Tottenham et al., 2009). For researchers concerned with using only those faces with the highest consensus, we recommend they use the available descriptive statistics for each model to make the best decision on model selection for their research.

Valence, intensity, and genuineness ratings support the finding of high emotion recognition accuracy. For valence, participants rated open-mouth happy faces more positively than neutral faces and closed-mouth happy faces, and closed-mouth happy faces were rated more positively than neutral faces. This is consistent with previous face databases that confirm neutral valence for neutral faces and positive valence for both closed and open-mouth happy faces (Conley et al., 2018; Langner et al., 2010; Tottenham et al., 2009). We also found that open-mouth happy faces were rated as more intense than both closed-mouth happy faces and neutral faces, and closed-mouth happy faces were rated as more intense than neutral faces. Overall, the TED stimuli were rated as moderately intense (*M* = 2.75) consistent with previous face databases that contain neutral and happy expressions (Langner et al., 2010; Palermo and Coltheart, 2004). For genuineness, open-mouth happy faces were rated as more genuine depictions of the intended expression than closed-mouth happy and neutral faces, and closed-mouth happy faces were rated as more genuine than neutral faces. These results are consistent with previous literature suggesting an increase in perceived genuineness for open-mouth happy expressions as compared to closed-mouth happy expressions (Chen et al., 2021; Langner et al., 2010; Wang et al., 2017).

Although accuracy, valence, intensity, and genuineness results demonstrate that overall, the TED face database consists of models displaying their intended emotions, there was variability among models in how accurately their emotional expressions were identified. The comprehensive TED face database is provided because of the need for representative stimuli of individuals of color; however, we also provide Supplementary Tables on the Open Science Framework with proportion correct, kappa, mean valence, mean intensity, and mean genuineness scores for each of the 805 stimuli that were validated, so that researchers can choose the subset of stimuli most appropriate for their research question.

There are a few limitations of the TED face database. The TED face database is limited in the range of emotional expressions represented. While it includes three emotional variations—neutral, open-mouth happiness, and closed-mouth happiness—it lacks other fundamental emotions such as anger, sadness, and disgust. This may restrict the types of research questions that can be posed regarding emotional expressions across diverse stimuli. Future research would benefit from the inclusion of ethnically diverse models exhibiting a broader spectrum of emotional expressions. Despite its limitations in emotional diversity, the TED face database remains one of the most diverse resources available for researchers investigating ethnically diverse stimuli. It offers a range of variations in other key dimensions, such as pose and gaze direction.

Moreover, the TED face database did not employ a fully crossed design, meaning that each face was not rated by an equal number of raters with the same demographic composition (i.e., an equal number of own-race and other-race raters). Although this would have been ideal, we were limited by the demographic composition of the undergraduate student population at Toronto Metropolitan University As a result, we are unable to draw conclusions about whether validation results would have differed for own-race vs. other-race raters. We recommend that researchers consider this limitation and the potential impact of the other-race effect, wherein individuals tend to recognize faces of their own race more accurately (Meissner and Brigham, 2001), when using the TED database in their own research. Future research should explore the influence of both face ethnicity and rater ethnicity on validation outcomes.

Another limitation of the TED face database is that it is demographically skewed in terms of age, ethnicity, and gender. Although there are models available of various ages, the TED face database mainly includes young adults. The number of models belonging to an age group outside of young adults is low; this may limit the potential questions researchers can ask. It will be important for future research to collect ethnically diverse models of various ages. Moreover, White faces are still over-represented in the database. However, the TED adds substantial diversity to existing face databases, by providing many representations of ethnically diverse models from various backgrounds displaying different poses. In the future, we hope that researchers will curate additional databases that increase the representation of other types of ethnically diverse individuals, including individuals from Indigenous populations. The gender composition of the TED face database is predominantly women due to the composition of the undergraduate psychology research pool where many of the models in the TED database were recruited, which reflects the gender imbalance of undergraduate programs in psychology (Gruber et al., 2021). Although this gender imbalance is not ideal, this database presents a valuable resource given that the documented bias in the literature is in the opposite direction—it relies on predominantly male face databases (Chen et al., 2021). We hope that the availability of the TED face database will help researchers address this bias in their future work; combining faces from the TED database with existing databases containing more male faces will allow researchers to implement research designs balanced on gender.

An advantage of the TED face database is that images were rated using a semi-forced choice design, allowing participants to choose across eight options (angry, disgust, fear, happy, neutral, sad, surprised, or “none of the above”) for each expression. Consistent with the NimStim (Tottenham et al., 2009) methods, the “none of the above” choice was included because strict forced choice tasks can inflate correct labeling. However, other research suggests that the subtle complexities of expressions may not fully be captured with this design and that a combination of forced choice, freely chosen, or spectrum (i.e., slightly happy, moderately happy, very happy) labels may be more appropriate for rating faces (Russell, 1994; Conley et al., 2018).

The TED face database addresses several limitations of available face databases. Previous research has relied heavily on White, male, or computer-generated faces. The TED directly addresses these limitations by substantially increasing the number of real models, who are predominantly women and from ethnically diverse backgrounds. Although existing face databases include some ethnic diversity, non-White individuals represented in those face databases are predominantly Black or East Asian (Conley et al., 2018; Lakshmi et al., 2021). The inclusion of multiple faces of South Asian, Southeast Asian, LatinX, and Middle Eastern descent may facilitate studies on ethnic minorities outside of Black and East Asian backgrounds. This is particularly important because these groups face prejudice and discrimination (e.g., French et al., 2013; Frey and Roysircar, 2006), yet remain largely understudied in the face perception literature. Thus, this may reduce the bias present within face databases and encourage research on the social invisibility experienced by women from underrepresented groups (Neel and Lassetter, 2019). This database may also facilitate research on the discrimination of individuals with intersecting race and gender identities (e.g., South Asian men). This is important as targets' race and gender can influence social perception (Chen et al., 2021), leading to divergent ratings on dimensions of trustworthiness, attractiveness, and dominance (Strachan et al., 2017; Zuckerman and Kieffer, 1994). By providing a set of real faces, the TED face database also addresses the reliance on computer-generated stimuli due to a lack of databases containing ethnically diverse faces with variations in pose, gaze, and emotional expression. The artificiality of computer-generated faces minimizes the variability within identity characteristics leading to altered perceptions of faces (Chen et al., 2021). Thus, researchers using TED can avoid such limitations by conducting experiments using ethnically diverse real faces.

Our main objective in developing the Toronto Ethnically Diverse (TED) face database was to create a large, ethnically diverse set of faces displaying different facial expressions and pose variations. The database contains 271 models with varying facial expressions, gaze directions, and poses available in color, offering researchers flexibility to combine TED stimuli with other facial expression databases. Diverse face databases of this nature may prove useful in examining psychological processes by providing representative stimuli that reflect the ethnicities of research participants and for testing questions specific to the effects of in-group vs. out-group membership on psychological processes. The TED face database is an open access tool that is available for free use to all academic researchers. By providing this tool, we hope to combat existing biases in the face perception literature and to contribute to advancing knowledge across the psychological literatures of face perception, impression formation, and intergroup relations.

## Data Availability

The datasets presented in this study can be found in online repositories. The names of the repository/repositories and accession number(s) can be found in the article/Supplementary material.
